# Film Deposition and Optical Properties of Cu-Based Metal Halide Cs_3_Cu_2_(I_1–*x*_Br_*x*_)_5_ Alloy via Mist Deposition

**DOI:** 10.1021/acsomega.4c09184

**Published:** 2025-03-10

**Authors:** Keisuke Watanabe, Kosuke Imai, Hiroyuki Nishinaka

**Affiliations:** † Department of Electronics, Kyoto Institute of Technology, Matsugasaki Sakyo-ku, Kyoto 606-8585, Japan; ‡ Faculty of Electrical Engineering and Electronics, Kyoto Institute of Technology, Matsugasaki Sakyo-ku, Kyoto 606-8585, Japan

## Abstract

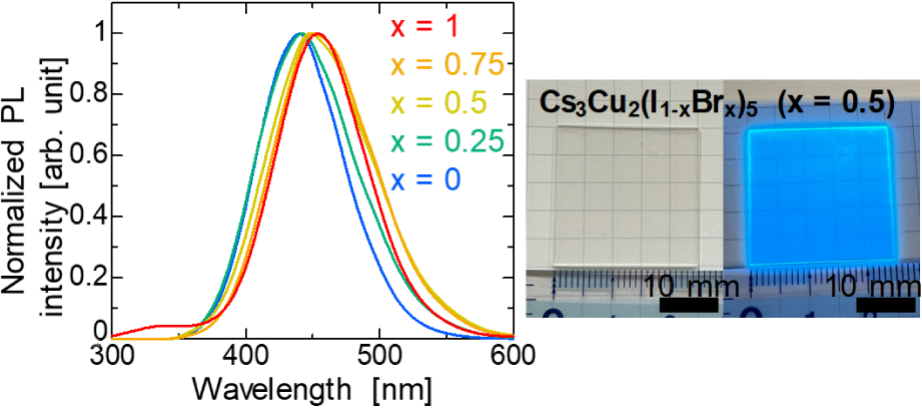

Cu-based metal halides, such as Cs_3_Cu_2_I_5_, have attracted significant attention as promising candidates for light-emitting diodes, photodetectors, and scintillators owing to their remarkable properties, including high photoluminescence quantum yield (PLQY), air stability, and nontoxicity. In particular, their emissive colors can be controlled by their halogen composition. However, a reliable technique for depositing halogen alloy thin films has not yet been established. Herein, we demonstrated the deposition of Cs_3_Cu_2_(I_1–*x*_Br_*x*_)_5_ alloy thin films via mist deposition, offering a scalable one-step method for Cu-based halide applications. X-ray diffraction and energy-dispersive X-ray spectroscopy revealed the successful formation of halogen alloy thin films with precise compositional control by varying the halide precursors. Additionally, alloy thin films formed via mist deposition exhibited high-coverage surfaces. Moreover, they exhibited blue photoluminescence peaks ranging from 440 to 456 nm under ultraviolet irradiation and a high PLQY of approximately 60%, reaching 92.4% for Cs_3_Cu_2_I_5_ thin film. Furthermore, the photoluminescence decay curve exhibited microsecond-order long PL lifetimes indicative of emission from self-trapped excitons. This study represents a significant breakthrough in the development of scalable, high-performance Cu-based metal halide alloy thin films for optical applications.

## 1 Introduction

All-inorganic metal halides, such as Pb-based halide perovskites, have attracted considerable attention because of their exceptional optical properties, such as adjustable bandgap, tunable emission spectra over the entire visible region, and high photoluminescence quantum yield (PLQY).^1,2^ These properties make them suitable for optoelectronic applications such as light-emitting diodes (LEDs),^3,4^ solar cells,^5^ and photodetectors.^6^ However, the presence of toxic metals, such as lead, poses risks to both human health and the environment, limiting their widespread application. Recent research has focused on developing lead-free alternatives, such as Sn-,^7,8^ Sb-,^9,10^ Bi-based halides ,^11,12^ and double perovskites.^13,14^ However, their inferior optical properties and instability in air hinder their practical application.

Among the lead-free metal halides, Cu-based halides^15−18^ have garnered significant interest because of their air stability, high PLQY, broadband emission, and large Stokes shifts. Specifically, Cs_3_Cu_2_I_5_^15^ has emerged as a promising material owing to its blue emission and exceptionally high PLQY exceeding 90%^15^ for single crystals and 80% for thin films.^16^ Thus, it offers considerable potential for application in LEDs,^15,19,20^ photodetectors,^21,22^ and scintillators.^23,24^ Moreover, similar to lead halide perovskites, their optical properties can be tailored by varying the halogen composition, as demonstrated in Cs_3_Cu_2_Br_5–*x*_I_*x*_^25^ and Cs_5_Cu_3_Cl_6_I_2_.^26^ However, although Cu-based halides exhibit excellent optical properties, most studies have focused on their application in nanocrystals^27,28^ and powders,^29,30^ and only a few have investigated their use in thin film deposition.^16^

This may be because depositing Cu-based halide thin films is challenging. Spin-coating, which is the primary facile method for obtaining Cu-based halide thin films, requires an inert atmosphere to form high-coverage thin films, limiting their large-area deposition. Zeng et al. achieved uniform thin films by dropping an antisolvent in a spin-coating process under an air atmosphere,^31^ and Sebastia-Luna et al. utilized thermal evaporation to obtain a thin film.^32^ However, these methods are complex and not ideal for mass production. Therefore, a simple high-coverage deposition method over a large area is crucial for Cu-based halide applications.

We propose mist deposition as a simple one-step method for forming large-area Cu-based metal halide thin films. This technique has been utilized for film deposition of Pb-based perovskite,^33−35^ double perovskite,^36^ and Cs_3_Cu_2_I_5_.^16,37^ In this study, we demonstrate the deposition of Cs_3_Cu_2_(I_1–*x*_Br_*x*_)_5_ alloy thin films using mist deposition and investigate their optical properties. Deposited alloy thin films cover a large area, and a high deposition rate of the submicron order per minute is achieved by adjusting the deposition conditions. The composition of Cs_3_Cu_2_(I_1–*x*_Br_*x*_)_5_ was also controlled by varying the ratio of the halide reactants in the precursor solution. The Cs_3_Cu_2_(I_1–*x*_Br_*x*_)_5_ alloy thin films exhibited blue emission from 440 to 456 nm under UV irradiation, which is comparable to previous reports for alloyed Cs_3_Cu_2_(I_1–*x*_Br_*x*_)_5_ powders.^25^ Notably, the Cs_3_Cu_2_I_5_ thin film showed a high PLQY exceeding 90%, which is, to our knowledge, the highest PLQY value in a thin film.

## 2 Experimental Method

Cesium iodide (CsI:99.9%), copper iodide (CuI:95%), cesium bromide (CsBr:99.9%), copper bromide (CuBr:95%), *N*,*N*-dimethylformamide (DMF:99.5%), and dimethyl sulfoxide (DMSO:99.0%) were purchased from Fujifilm Wako Chemicals. The precursor solution was prepared by dissolving stoichiometric amounts of CsI, CuI, CsBr, and CuBr in a mixture of DMF and DMSO (4:1, v/v). The total concentration of the precursor solution was 0.025 mol/L, consisting of 0.015 mol/L of CsX and 0.010 mol/L of CuX (X = I, Br) to set the Cs:Cu ratio in the solution to 3:2. Alloy thin films were formed by varying *x* every 0.25. For example, when *x* = 0.25, the composition is Cs_3_Cu_2_I_3.75_Br_1.25_. The concentrations of each precursor are summarized in Table 1.

**Table 1:** Concentration of CsI, CuI, CsBr, and CuBr in the Precursor Solution^a^

*x*	CsI [mol/L]	CuI [mol/L]	CsBr [mol/L]	CuBr [mol/L]
0	1.5 × 10^–2^	1.0 × 10^–2^	-	-
0.25	1.125 × 10^–2^	7.5 × 10^–3^	3.75 × 10^–3^	2.5 × 10^–3^
0.5	7.5 × 10^–3^	5.0 × 10^–3^	7.5 × 10^–3^	5.0 × 10^–3^
0.75	3.75 × 10^–3^	2.5 × 10^–3^	1.125 × 10^–2^	7.5 × 10^–3^
1	-	-	1.5 × 10^–2^	1.0 × 10^–2^

a The total concentration of all precursors in solution was adjusted to 0.025 mol/L.

Cs_3_Cu_2_(I_1–*x*_Br_*x*_)_5_ alloy thin films were prepared by mist deposition. In this technique, the precursor solution was atomized into mist particles by using 2.4 MHz ultrasonic vibrations. The resulting mist particle size, approximately 3 μm, was suitable for transport by the carrier gas. The generated mist particles were transferred to a heated nozzle with a rectangular aperture (1 × 50 mm^2^) using nitrogen (N_2_) gas as the carrier gas at a flow rate of 9 L/min. A curtain-like mist flow was formed and continuously supplied to the preheated quartz substrate maintained at 140 °C. As the mist flow came into contact with the preheated substrate, the precursor mist evaporated and formed thin films.

The crystallinity and lattice parameters of the Cs_3_Cu_2_(I_1–*x*_Br_*x*_)_5_ alloy thin films were evaluated by using X-ray diffraction (XRD) 2θ–ω scan profiles (D8 DISCOVER, Bruker). The surface morphologies of the alloy thin films were observed using field-emission scanning electron microscopy (FESEM) (S-5200, Hitachi). The film thickness was measured by stylus profilometry (Dektak XT-S, Bruker). The compositions of the alloy thin films were analyzed by SEM (TM3030Plus, Hitachi) coupled with energy-dispersive X-ray spectroscopy (EDX) (x-stream-2, Oxford Instruments). To evaluate the optical properties of the Cs_3_Cu_2_(I_1–*x*_Br_*x*_)_5_ alloy thin films, PL and photoluminescence excitation (PLE) spectroscopies were performed by using a fluorescence spectrometer (F-2700, Hitachi). PLQY measurements were conducted using an absolute PL quantum yield spectrometer (Quantaurus-QY C11347, Hamamatsu Photonics). This apparatus, equipped with an integrating sphere, was used to measure the emission spectra of both the reference substrate and the formed thin films. Absolute PLQYs were then determined from the emission intensity and absorbed excitation light. PL decay curve analysis was conducted using a fluorescence lifetime spectrometer (Quantaurus-Tau C11367, Hamamatsu Photonics).

## 3 Result and Discussion

First, we performed a compositional analysis of the Cs_3_Cu_2_(I_1–*x*_Br_*x*_)_5_ alloy thin films using EDX. Table 2 summarizes the composition concentration ratios of Cs, Cu, I, and Br in the alloy thin films as measured by EDX. When we examined the composition ratio with Cs as the reference, for example, when *x* = 0, the Cs:Cu:I ratio was 3:3.4:5.1. Similar values were observed for other *x* values, with excess Cu consistently present. In contrast, the halogens were nearly stoichiometric with respect to Cs. When Cs is incorporated in a stoichiometric ratio, it is possible that Cu is incorporated in the form of Cu ions or oxides such as Cu_2_O or CuO. This is because the halogens in CuI and CuBr desorbed and evaporated at a relatively low temperature,^38^ which can be explained by the excess Cu content. Although O peaks were also observed in the EDX analysis, they included contributions from the substrate (SiO_2_), making the quantitative evaluation challenging. Further assessments are required for this evaluation.

**Table 2:** Composition Concentration of Cs, Cu, I, and Br in Cs_3_Cu_2_(I_1–*x*_Br_*x*_)_5_ Alloy Thin Films^a^

*x*	Cs	Cu	I	Br
0	25.97	29.47	44.56	-
0.25	26.10	27.84	33.68	12.39
0.5	26.54	27.61	20.48	25.38
0.75	25.55	27.46	9.88	37.11
1	27.31	25.57	-	47.12

a The ratio of Cs:Cu:(I + Br) was nearly 3:3:5 for all *x v*alues, with excess Cu presented.

**Figure 1:**
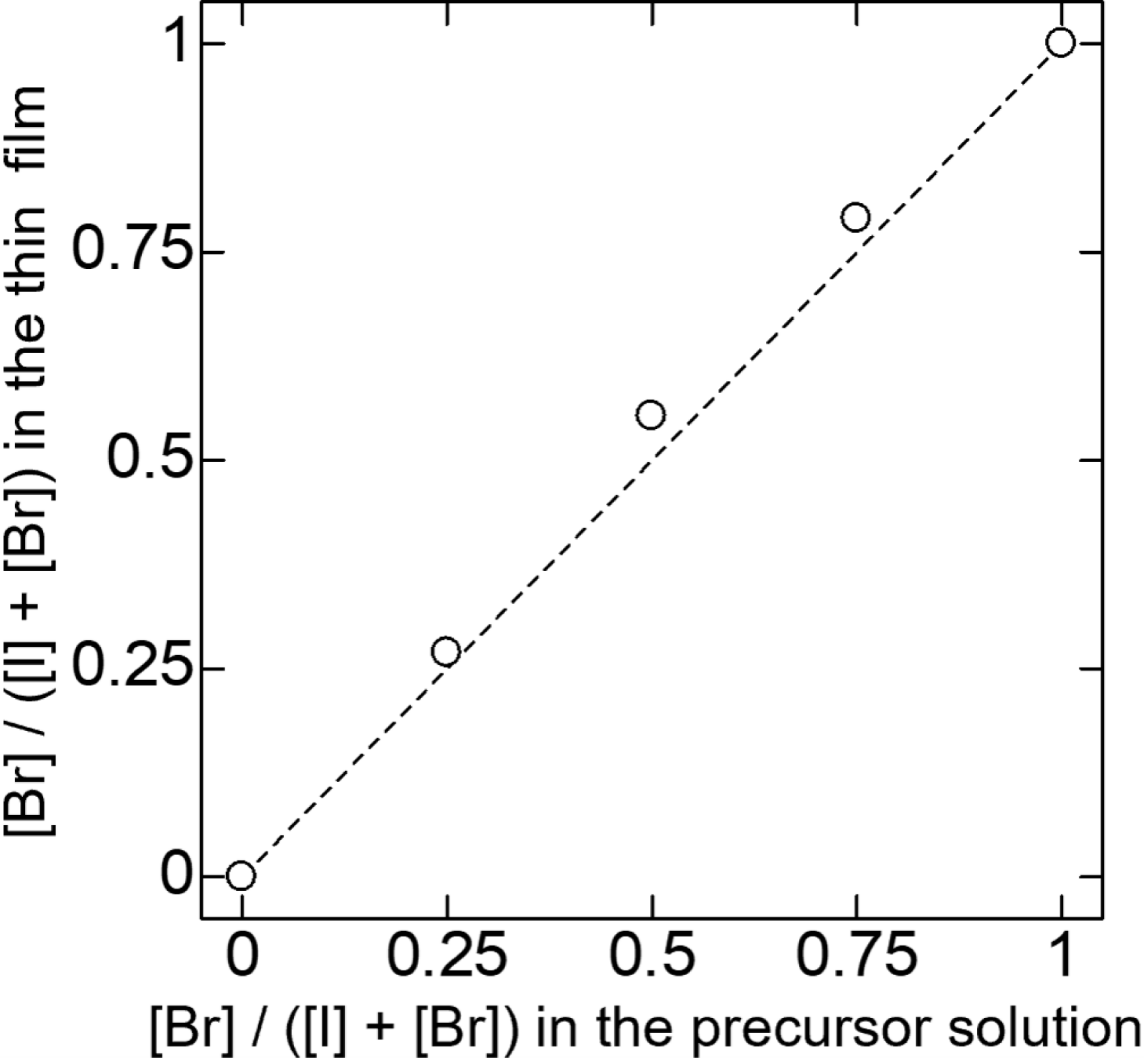
Relationship between the Br concentration in the precursor solution and that in the thin films. The Br concentration in the alloy thin films linearly increases and consistent with that in precursor solution.

Additionally, Figure 1 shows the [Br]/([I] + [Br]) ratio in the thin film compared to that in the solution. The Br composition ratio was almost identical between that of the solution and the thin films. These demonstrations using mist deposition allowed the composition ratio of I to Br in the thin film to be controlled almost precisely, in line with the solution.

**Figure 2:**
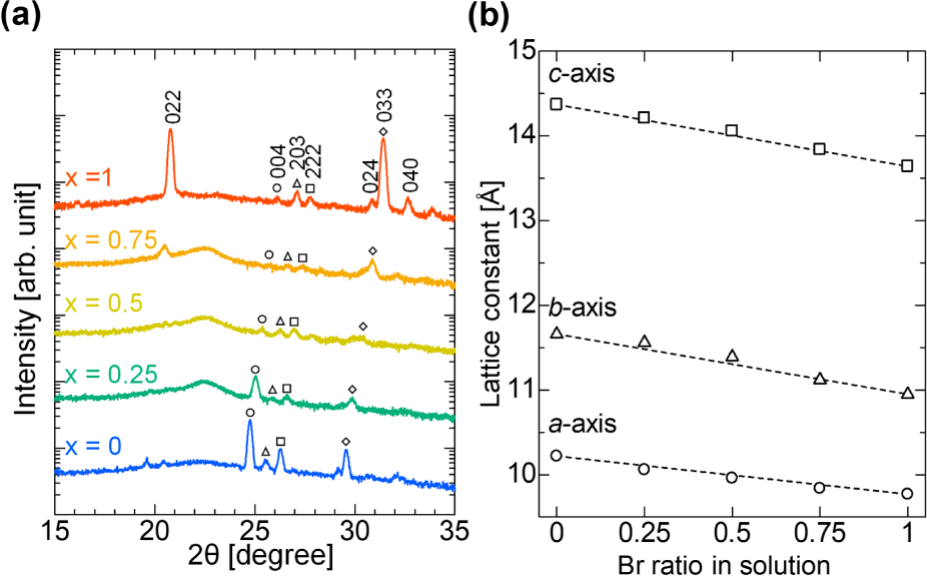
(a) XRD 2θ–ω scan profile of the Cs_3_Cu_2_(I_1–*x*_Br_*x*_)_5_ alloy thin films formed via mist deposition. Several diffraction peaks were observed for all samples. Each peak shifted to higher angles with increasing Br content in thin film. (b) Lattice constant including *a*-, *b*-, and *c*-axis of Cs_3_Cu_2_(I_1–*x*_Br_*x*_)_5_ alloy thin films calculated from XRD 2θ–ω peaks. We calculated these lattice parameters using the peak of (004), (222), and (033). All lattice parameters decreased linearly as the Br content in the thin films increases.

XRD measurements were performed to verify the crystal phase and lattice parameters of the Cs_3_Cu_2_(I_1–*x*_Br_*x*_)_5_ alloy thin films. Figure 2a presents the XRD 2θ–ω scan profiles of the Cs_3_Cu_2_(I_1–*x*_Br_*x*_)_5_ alloy thin films. Distinct diffraction peaks were observed for all samples, including those with varying alloy compositions. These peaks shift to higher angles with increasing Br content in the film, suggesting the formation of Cs_3_Cu_2_(I_1–*x*_Br_*x*_)_5_ alloy films and a decrease in the lattice parameters as I is replaced with Br. This peak shift is consistent with a previous report on nanocrystals.^18^ However, in the alloy compositions, the peak intensity was reduced compared to that of the pure Cs_3_Cu_2_I_5_ or Cs_3_Cu_2_Br_5_ thin films, which can be attributed to degradation of the crystalline quality due to halogen alloying.

The lattice parameters of the alloy compositions were calculated from the XRD peaks of the Cs_3_Cu_2_(I_1–*x*_Br_*x*_)_5_ alloy films. Both Cs_3_Cu_2_I_5_ and Cs_3_Cu_2_Br_5_ belong to the orthorhombic phase with a space group of *Pnma*. We utilized the (004), (222), and (033) peaks to calculate the lattice parameters. The calculated lattice constants are presented in Figure 2b. The lattice parameters of Cs_3_Cu_2_I_5_ and Cs_3_Cu_2_Br_5_ were consistent with previous reports.^15,25^ In the alloy compositions, a near-linear decrease in the *a*-, *b*-, and *c*-axis was observed with an increasing Br ratio. This suggests that the Cs_3_Cu_2_(I_1–*x*_Br_*x*_)_5_ alloy thin films closely follow Vegard’s Law,^39^ similar to their powders.^25^ These results indicate that the thin films formed via mist deposition are alloy thin films with substituted halogens and not a mixture of the individual phases of Cs_3_Cu_2_I_5_ and Cs_3_Cu_2_Br_5_.

**Figure 3:**
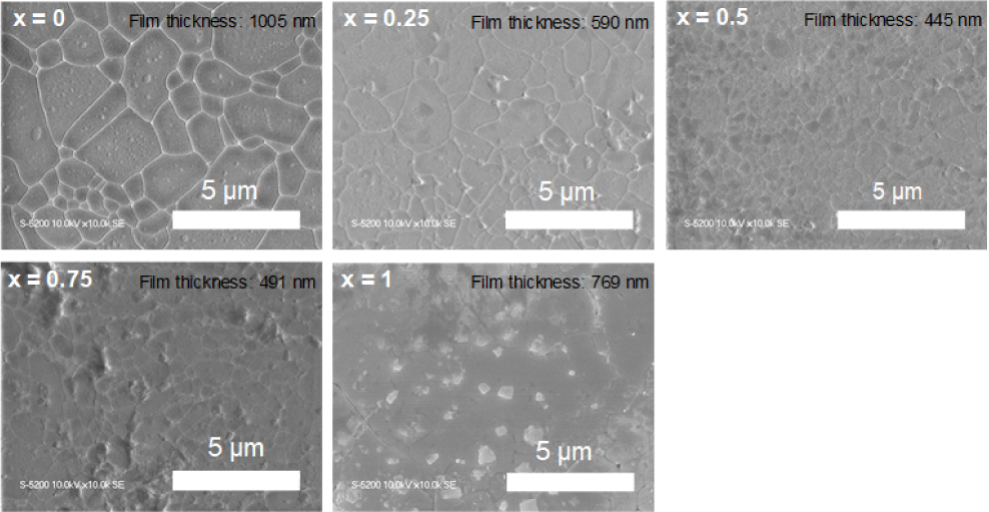
Top-view scanning electron microscopy (SEM) images of Cs_3_Cu_2_(I_1–*x*_Br_*x*_)_5_ alloy thin films with *x* = 0, 0.25. 0.5, 0.75, and 1. The films show systematic morphological evolution from large polygonal grains (*x* = 0) to finer grain structure (*x* = 0.5). Film thicknesses are shown in the top-right of each image: 1005 nm (*x* = 0), 590 nm (*x* = 0.25), 445 nm (*x* = 0.5), 491 nm (*x* = 0.75), and 769 nm (*x* = 1).

Figure 3 shows SEM images of the Cs_3_Cu_2_(I_1–*x*_Br_*x*_)_5_ alloy thin films. In a previous study, we demonstrated high-coverage Cs_3_Cu_2_I_5_ thin films using mist deposition.^16^ As shown in Figure 3, we successfully formed high-coverage thin films of the Cs_3_Cu_2_(I_1–*x*_Br_*x*_)_5_ alloy. This is attributed to the continuous supply of precursor mist, which promotes the growth of grains and coverage of the entire film. The pure iodide film (*x* = 0) exhibits large, well-defined polygonal grains in the micrometer range. As *x* increases, the grain size decreases as the surface morphology becomes progressively rougher and more textured. This is because of the more frequent generation of crystal nuclei by halogen alloying. At *x* = 1 (pure bromide), the thin film exhibited a more heterogeneous surface. In this study, the same deposition conditions based on Cs_3_Cu_2_I_5_ formation were used for all composition ratios. These conditions may not be suitable for depositing Cs_3_Cu_2_Br_5_ thin films. In mist deposition, numerous parameters such as the precursor solution concentration, solvent mixing ratio, carrier gas flow rate, and substrate heating temperature are involved, making it challenging to determine a single critical factor. On the other hand, in this study, the precursor solution concentration appears to be the most influential parameter that affects the deposition conditions. Each precursor exhibits different solubilities in DMSO and DMF. For instance, the CsI and CuI mixture with a 3:2 molar ratio dissolves up to 2.8 mol/L (CsI: 1.68 mol/L, CuI: 1.12 mol/L) in a DMF/DMSO mixed solvent under the present conditions, whereas the CsBr and CuBr mixture dissolves only up to 0.5 mol/L (CsBr: 0.3 mol/L, CuBr: 0.2 mol/L). This difference in solubility likely influences the time it takes for mist droplets to reach the substrate, evaporate, and attain the saturation point, where crystal nuclei form. Therefore, appropriately adjusting the deposition conditions, such as the precursor solution concentration, for each composition improves the quality of Cs_3_Cu_2_Br_5_ thin films. We presented the thickness of Cs_3_Cu_2_(I_1–*x*_Br_*x*_)_5_ alloy thin films in the top right of Figure 3. In the pure Cs_3_Cu_2_I_5_ and Cs_3_Cu_2_Br_5_ phases for *x* = 0 and 1, the thickness of thin films reached 1005 and 769 nm, respectively. In this study, heated substrates were transferred at a speed of 2.5 mm/min, and thin film deposition was completed in a few minutes. This suggests that a high deposition rate was achieved. In contrast, thinner films with an average thickness of approximately 500 nm were obtained for *x* = 0.25, 0.5, and 0.75. This trend highlights that halogen alloying leads to a slower deposition rate owing to the difference in the growth mechanism between the pure and alloying phases.

**Figure 4:**
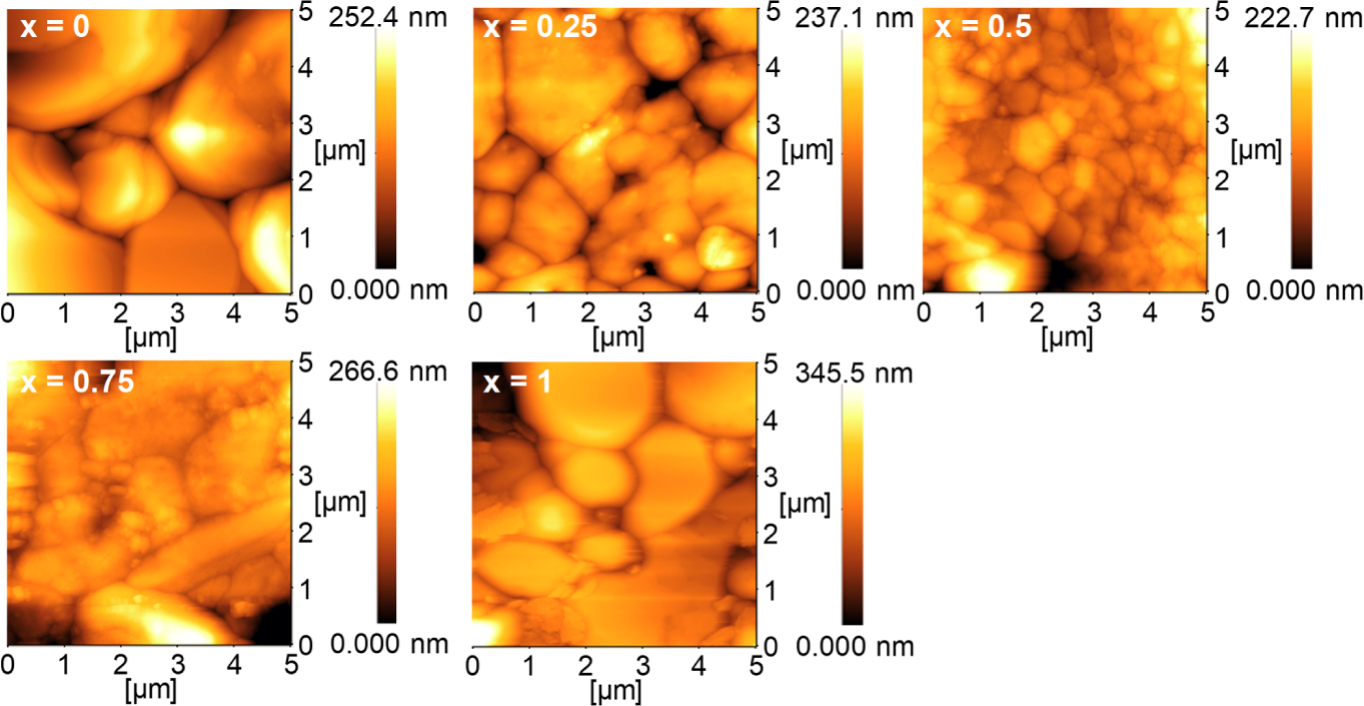
AFM images of Cs_3_Cu_2_(I_1–*x*_Br_*x*_)_5_ alloy thin films with *x* = 0, 0.25, 0.5, 0.75, and 1. The maximum height of thin films increased, and the surface of alloy thin films became rougher and textured as the Br content in thin films increased.

**Figure 5:**
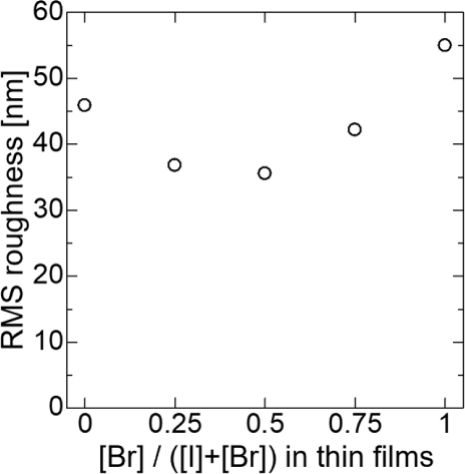
RMS roughness of Cs_3_Cu_2_(I_1–*x*_Br_*x*_)_5_ alloy thin films measured via AFM measurement. The RMS roughness exhibited a range from 35.6 to 55.0 nm, with the smallest value observed at *x* = 0.5, which was composed of finer grains.

To evaluate the surface flatness of the thin films quantitatively, AFM measurements were performed, and the RMS roughness was calculated. Figure 4 shows AFM images of Cs_3_Cu_2_(I_1–*x*_Br_*x*_)_5_. As suggested by the FESEM images, the surface grain size decreased as the Br content increased, exhibiting a fine-grain morphology at *x* = 0.5. Additionally, the maximum height for thin films was observed in pure Cs_3_Cu_2_ Br_5_ for *x* = 1, indicating a rougher thin film surface. Figure 5 presents a plot of the RMS roughness values obtained for each I–Br halogen composition from AFM measurements. The RMS roughness ranged from 35.6 to 55.0 nm. Considering that the film thickness for each composition is approximately several hundred nanometers or a micrometer, these RMS roughness values indicate the thin films are sufficiently flat. Therefore, flatter thin films were achieved in this study using a simple technique.

**Figure 6:**
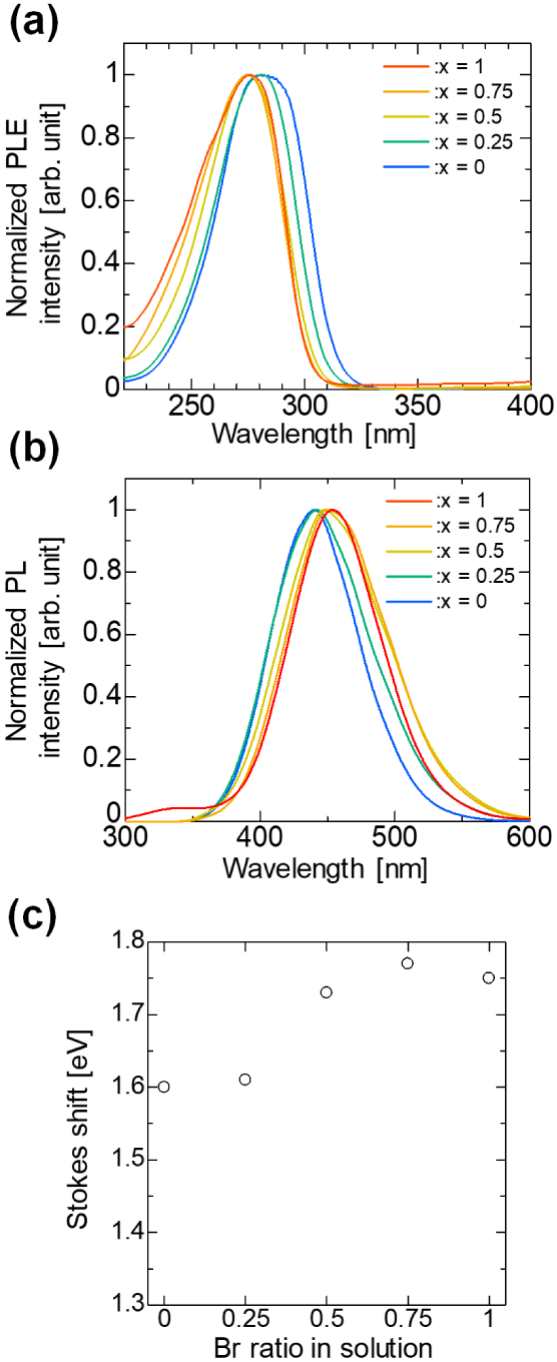
(a) PLE spectra of Cs_3_Cu_2_(I_1–*x*_Br_*x*_)_5_ thin films monitored at each PL peaks. PLE peaks of alloy thin films exhibit a systematic blue shift as the Br content in the thin films increases. (b) PL spectra of Cs_3_Cu_2_(I_1–*x*_Br_*x*_)_5_ thin films excited at wavelength of 290 nm. PL peaks of alloy thin films are red-shifted as the Br content in the thin films increases. (c) Stokes shift, difference between the peaks of PL and PLE, of Cs_3_Cu_2_(I_1–*x*_Br_*x*_)_5_ alloy thin films. The Stokes shift of alloy thin films was obtained in the range from 1.60 to 1.77 eV and generally increases with the Br content.

Next, we investigated the optical properties of the Cs_3_Cu_2_(I_1–*x*_Br_*x*_)_5_ alloy thin films. Figure 6a shows the PLE spectra of the Cs_3_Cu_2_(I_1–*x*_Br_*x*_)_5_ alloy thin films. The PLE spectra reveal a small and systematic blue shift of the excitation peaks with increasing Br content (*x*). This shift was attributed to the widening of the bandgap as Br substituted for I in the crystal structure. Figure 6b shows the PL spectra of the Cs_3_Cu_2_(I_1–*x*_Br_*x*_)_5_ alloy thin films. As shown in Figure 6b, the PL spectra exhibited a trend contrasting with the PLE results. As the Br content increased, the emission peaks exhibited a systematic red shift. These observations aligned with the previously reported emission wavelengths for Cs_3_Cu_2_I_5_ (440 nm) and Cs_3_Cu_2_Br_5_ (460 nm).^25^ The data show a systematic shift between these two extremes as the Br content changes, indicating tunable emissions across this range. The Stokes shift, calculated as the energy difference between the excitation and emission peaks, generally increased with increasing Br content (Figure 6c). This large Stokes shift is due to the emission from the self-trapped exciton.^40^ Overall, these results demonstrate that the optical properties of Cs_3_Cu_2_(I_1–*x*_Br_*x*_)_5_ thin films can be tuned by adjusting the Br:I ratio. These PL/PLE results are consistent with previous powder reports.^25^

**Figure 7:**
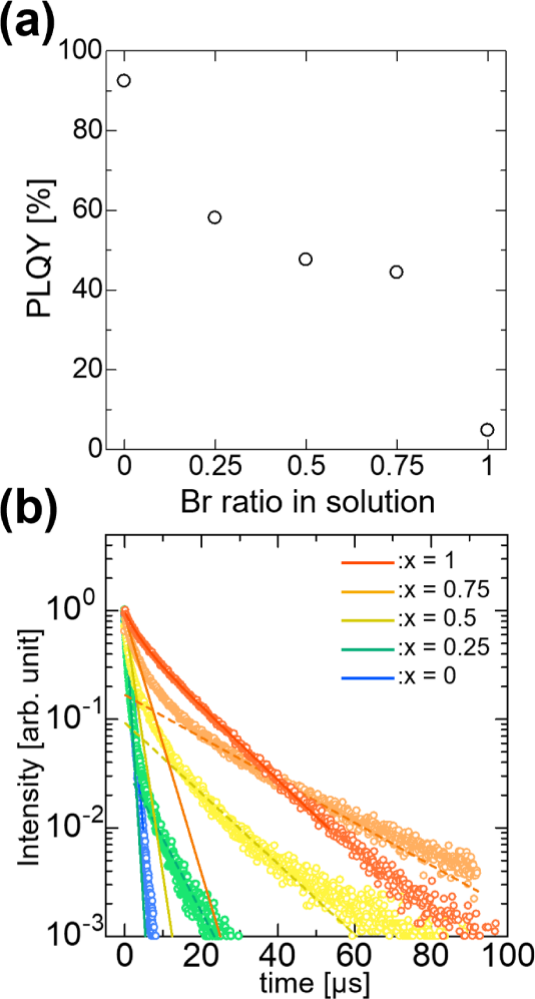
(a) PLQY of Cs_3_Cu_2_(I_1–*x*_Br_*x*_)_5_ alloy thin films observed under 290 nm excitation. A PLQY of 92.4% was obtained for Cs_3_Cu_2_I_5_ thin films (*x* = 0), and it decreased as the Br content increased. (b) PL decay curve measured under 280 nm excitation of Cs_3_Cu_2_(I_1–*x*_Br_*x*_)_5_ alloy thin films. The PL decay curve of the alloy thin films exhibited microsecond-order monoexponential decay in the pure phase (*x* = 0 and *x* = 1) and biexponential decay in the alloy phase (*x* = 0.25, 0.5, and 0.75).

Figure 7a shows the PLQY of the Cs_3_Cu_2_(I_1–*x*_Br_*x*_)_5_ alloy thin films. A high PLQY of 92.4% was achieved for the Cs_3_Cu_2_I_5_ thin films (*x* = 0). Notably, this high PLQY is comparable to those of single crystals, powders, and nanocrystals. Typically, thin films exhibit a lower PLQY than that of single crystals. For example, Jun et al. reported a PLQY of approximately 90% for the single crystals, whereas a lower PLQY of about 60% was obtained for the thin films.^15^ The high PLQY observed in this study is due to the enhanced crystallinity of the thin films. As Br content increased, there was a notable decrease in PLQY, with the lowest value being 4.8% for the Cs_3_Cu_2_Br_5_ thin film. A similar tendency was reported in a previous powder report.^25^ In that study, Cs_3_Cu_2_Br_5_ powder showed a PLQY of 50%. We speculate that the lower PLQY observed in thin films than in single crystals or powders is due to their air sensitivity.

To investigate the PL lifetimes of the Cs_3_Cu_2_(I_1–*x*_Br_*x*_)_5_ alloy thin films, we conducted PL decay curve measurements at room temperature. Figure 7b shows the PL decay curves of the Cs_3_Cu_2_(I_1–*x*_Br_*x*_)_5_ alloy thin films. All of the compositions exhibited microsecond-order PL decay. The PL decay curves of the Cs_3_Cu_2_I_5_ (*x* = 0) and the Cs_3_Cu_2_Br_5_ (*x* = 1) thin films decrease monoexponentially, suggesting both Cs_3_Cu_2_I_5_ and Cs_3_Cu_2_Br_5_ have one decay component, which is consistent with a previous study.^18^ By contrast, the PL decay curves of the alloy thin films (*x* = 0.25, 0.5, 0.75) decrease biexponentially, indicating the existence of two decay components. The slow component becomes more dominant with an increasing Br content in the alloy film. Therefore, it is considered that these two decay components are related to Cs_3_Cu_2_I_5_ and Cs_3_Cu_2_Br_5_, respectively. However, more detailed investigations are required to precisely understand the emission dynamics of Cu-based metal halide alloy thin films.

We also estimated the PL lifetimes of the Cs_3_Cu_2_(I_1–*x*_Br_*x*_)_5_ alloy thin films. As shown in Table 3, the PL lifetimes of Cs_3_Cu_2_I_5_ and Cs_3_Cu_2_Br_5_ are 1.032 and 11.34 μs, respectively, consistent with a previous report on nanocrystals.^18^ In the alloy phase, each PL lifetime component increased with Br content. The long PL lifetimes of the order of microseconds suggest emission from self-trapped excitons in the Cu-based metal halides.^40^ In our previous study, we deposited the Cs_3_Cu_2_I_5_ thin films at various substrate temperatures using mist deposition.^16^ In that report, the thin films formed at 180 °C exhibited poor surface morphology with low surface coverage and the presence of voids. On the other hand, their optical properties, including PL, PLQY, and PL lifetime, were comparable to those of the thin films with improved surfaces obtained at optimal substrate temperatures. This suggests that the surface morphology of the thin films had a minimal effect on the optical properties of Cs_3_Cu_2_I_5_. Therefore, in the present study, we focused exclusively on the compositional effects on the optical properties and assumed that the influence of thin film is negligible.

**Table 3:** PL Lifetime, Obtained from the PL Decay Curve Fitting, and the Radiative and Nonradiative Rate Constants, Calculated from PLQYs and PL Lifetimes of Cs_3_Cu_2_(I_1–*x*_Br_*x*_)_5_ Alloy Thin Films^a^

*x*	τ_1_ [μs]	τ_2_ [μs]	*k*_r_	*k*_nr_
0	1.032	-	9.0 × 10^5^	7.4 × 10^4^
0.25	0.7740	6.480	-	-
0.5	1.844	9.611	-	-
0.75	3.677	22.15	-	-
1	11.34	-	4.2 × 10^3^	8.4 × 10^4^

a A single PL lifetime was observed in the pure phase of Cs_3_Cu_2_I_5_ (*x* = 0) and Cs_3_Cu_2_Br_5_ (*x* = 1). In the alloy phase of *x* = 0.25, 0.5, and 0.75, two PL lifetimes corresponding to two decay components were obtained. The radiative rate constant decreased from 9.0 × 10^5^ to 4.2 × 10^3^, while the non-radiative rate constant exhibited 10^4^ order values for Cs_3_Cu_2_I_5_ and Cs_3_Cu_2_Br_5_.

Additionally, we calculated the rate constants of the radiative and nonradiative transitions for pure Cs_3_Cu_2_I_5_ (*x* = 0) and Cs_3_Cu_2_Br_5_ (*x* = 1), which exhibited monoexponential decay, from measured PLQY and PL lifetimes using the following relations:^41^
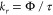

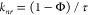
where Φ represents the PLQY, and τ represents the PL lifetime. Table 3 presents the calculated rate constants for the Cs_3_Cu_2_I_5_ and Cs_3_Cu_2_Br_5_ thin films. For Cs_3_Cu_2_I_5_ (*x* = 0), the radiative rate constant is ten times greater than the nonradiative rate constant, indicating that radiative transitions were dominant. Conversely, for Cs_3_Cu_2_Br_5_ (*x* = 1), the nonradiative rate constant was higher, suggesting that nonradiative transitions were dominant under this composition.

## 4 Conclusion

In this study, we demonstrate the deposition of Cs_3_Cu_2_(I_1–*x*_Br_*x*_)_5_ alloy thin films using mist deposition and investigate their optical properties. The alloy thin films formed by mist deposition exhibited smooth surfaces. The halogen ratio in the thin films could be controlled by adjusting the ratio of the precursor solution. The alloy thin films displayed blue emission, with peaks ranging from 440 to 456 nm. The PLQY showed over 90% in Cs_3_Cu_2_I_5_ and decreased with increasing Br ratio. Our research provides a novel approach and valuable insights into the deposition of Cu-based metal halide alloy thin films for optical applications.
